# Management of a centenarian who underwent emergency laparoscopic cholecystectomy under general anesthesia with subcostal transversus abdominis plane block

**DOI:** 10.1186/s40981-016-0050-9

**Published:** 2016-09-23

**Authors:** Yoshiyuki Nagamine, Kohei Godai, Hiroshi Oki, Yuichi Kanmura

**Affiliations:** 1 Department of Anesthesiology, Kagoshima Prefectural Ohshima Hospital, 18-1 Nazemanadu, Amami, Kagoshima 894-0015 Japan; 2 Department of Anesthesiology and Critical Care Medicine, Graduate School of Medical and Dental Sciences, Kagoshima University, 8-35-1 Sakuragaoka, Kagoshima, 890-8520 Japan

**Keywords:** Centenarian, Laparoscopic cholecystectomy, Transversus abdominis plane block

## Abstract

The anesthetic management of centenarians is challenging, since they have loss of functional reserve in all organs. The mortality rate of 25 % is reported in patients over 100 years old who underwent emergency surgery. The transversus abdominis plane block has been shown to provide effective analgesia in laparoscopic cholecystectomy. A 101-year-old woman was diagnosed with grade I (mild) acute cholecystitis with gallstones. An emergency laparoscopic cholecystectomy was scheduled. The patient had a history of hypertension. The patient’s laboratory data showed that she had mild coagulopathy, anemia, thrombocytopenia, and decreased renal function. After induction of general anesthesia, an ultrasound-guided, bilateral subcostal transversus abdominis plane block was performed. Her postoperative course was uneventful. Using the preoperative subcostal transversus abdominis plane block, we were able to avoid hemodynamic instability and to reduce opioid dosage in a centenarian who underwent emergency laparoscopic cholecystectomy under general anesthesia.

## Background

The incidence of gallstones is known to increase with age [1]. Laparoscopic cholecystectomy (LC) is reported to be safe and feasible in patients older than 80 years [2]. The number of centenarians who undergo LC is, therefore, likely to continue increasing. The updated Tokyo Guidelines 2013 for acute cholangitis and acute cholecystitis recommend emergency LC for patients with grade I (mild) and grade II (moderate) acute cholecystitis [3]. Delayed elective cholecystectomy after gallbladder drainage should be performed in patients with grade III (severe) acute cholecystitis.

The anesthetic management of centenarians is challenging, since they have loss of functional reserve in all organs, decreased functional capacity, and imbalance of homeostasis. The mortality rate of 25 % is reported in patients over 100 years old who underwent emergency surgery [4]. Although intraoperative and postoperative analgesia in patients undergoing LC can be achieved with intravenous opioid or thoracic epidural analgesia, there are concerns about opioid-related side effects and hemodynamic instability in the elderly patients [5]. The transversus abdominis plane block (TAPB) has been shown to provide effective analgesia in LC [6]. TAPB seems to be a preferable analgesia in the elderly patients undergoing LC. We here report an anesthetic management of a centenarian patient who underwent LC using TAPB.

## Case presentation

A 101-year-old woman (145 cm, 45.2 kg) was diagnosed with grade I (mild) acute cholecystitis with gallstones. Although she had a 2-day history of right upper abdominal quadrant pain, Murphy’s sign was not seen. She had a fever (37.7 °C) and elevated C-reactive protein (24.3 mg/dl), white blood cell count (15,520/mm^3^), total bilirubin (1.4 mg/dl), and blood urea nitrogen (28.2 mg/dl). Gallbladder distention, pericholecystic fat stranding, and gallbladder wall thickening were seen on computed tomography. She did not show any signs of organ dysfunction or grade II (moderate) acute cholecystitis. Markedly elevated white blood cell count (>18,000/mm^3^), palpable tender mass in the right upper abdominal quadrant, duration of complaints >72 h, and marked local inflammation (gangrenous cholecystitis, pericholecystic abscess, hepatic abscess, biliary peritonitis, emphysematous cholecystitis) are considered as signs of grade II (moderate) acute cholecystitis [7]. She was able to walk with a cane without help. She did not need any supports with eating, bathing, dressing, and toileting. An emergency LC was scheduled. Preoperative gallbladder drainage was not performed. Pre-anesthetic examination of the patient showed that she was taking telmisartan 30 mg and losartan 50 mg daily for hypertension. The patient’s laboratory data showed that she had mild coagulopathy (prothrombin time international normalized ratio of 1.46 and activated partial thromboplastin time of 42 s), anemia (hemoglobin concentration of 11.8 g/dl), thrombocytopenia (platelet count of 103,000/μl), and decreased renal function (estimated glomerular filtration rate of 30.7 ml/min/1.73 m^2^). We considered that the patient had an American Society of Anesthesiologists physical status of II. We planned general anesthesia with TAPB to avoid hemodynamic instability and to reduce opioid dosage.

General anesthesia was induced with midazolam (3 mg) and fentanyl (200 μg). Rocuronium (30 mg) was administered to facilitate tracheal intubation. After induction of general anesthesia, we performed ultrasound-guided, bilateral subcostal TAPB. The patient had thin abdominal muscles. We injected 0.3 % ropivacaine 25 ml on each side (a total of 50 ml). General anesthesia was maintained with desflurane 3 % and remifentanil 0.05–0.15 μg/kg/min. Phenylephrine 5–12 μg/min was used to maintain mean blood pressure of 65 mmHg. A total of fentanyl 250 μg was used during surgery. At the end of the procedure, 680 mg of acetaminophen was given intravenously for postoperative analgesia. Residual neuromuscular block was antagonized with sugammadex (2 mg/kg) after confirming a train of four counts of two. After the extubation of the trachea, she had no pain at rest and on coughing (visual analogue scale of 0.0 and 0.0 mm, at rest and on coughing, respectively). Loxoprofen 60 mg was used for the first time postoperatively at 12 h after surgery. Oral intake was also resumed 12 h after surgery. Her postoperative course was uneventful. The patient was discharged from the hospital on postoperative day 6.

### Discussion

This case report shows that TAPB provided adequate postoperative analgesia with sparing opioid dosage and avoiding hemodynamic instability in a centenarian patient who underwent LC.

LC is a safe procedure in the extremely elderly patients [2]. Four types of postoperative analgesia (intravenous opioid, thoracic epidural analgesia, TAPB, and port-site infiltration of local anesthetics) are mainly used in LC. Each of the four analgesia methods has its advantages and disadvantages. Intravenous opioid is the preferable choice in patients with severe coagulopathy or thrombocytopenia. Intravenous opioid, however, has catastrophic side effects like respiratory depression [8]. Since the elderly patients are prone to opioid-related side effects (respiratory depression, nausea, and sedation), anesthesiologists tend to avoid aggressive use of postoperative opioids [9]. Thoracic epidural analgesia is effective for both somatic pain and visceral pain. The major drawback of thoracic epidural analgesia is its sympathetic blockade effect which leads to hypotension and bradycardia. TAPB can be performed in patients with mild coagulopathy like our patient, since bleeding after TAPB is easy to stop with compression. Although TAPB is not effective for visceral pain, the authors considered that TAPB is the preferred analgesic choice for centenarian patients undergoing LC because of its analgesic effects and less side effects than intravenous opioid or thoracic epidural analgesia. There are several TAPB techniques, including lateral TAPB, subcostal TAPB, and posterior TAPB [10]. Port-site infiltration of local anesthetics is the easiest way of postoperative analgesia. Although lateral TAPB was reported to be equivalent to port-site infiltration of local anesthetics, subcostal TAPB provides superior postoperative analgesia and reduces opioid requirement following LC than port-site infiltration [11, 12]. Compared with lateral TAPB, subcostal TAPB provides better postoperative analgesia [13]. In addition, preoperative subcostal TAPB may be able to reduce intraoperative opioids, and this is the reason why we selected preoperative subcostal TAPB.

The emergency surgery is known to be a risk factor for poor outcomes in elderly patients [14]. The mortality at postoperative 30 days among centenarian patients undergoing emergency surgery was reportedly 25 % [4]. Since intraoperative hypotension is associated with a 30-day mortality, phenylephrine 5–12 μg/min was used in the patient to avoid hypotension [15]. The authors used a total of 150 mg ropivacaine (3.3 mg/kg). The dosage is above the maximum recommended dose (3 mg/kg) [16]. The patient did not show any signs of local anesthetic systemic toxicity. Loxoprofen was used in the patient despite her having decreased renal function. The use of NSAIDS in fragile elderly, often hypovolemic patients with limited renal functional reserve, is not recommended [17]. We should have chosen acetaminophen instead of non-steroidal anti-inflammatory drugs in the very elderly patients. The patient was successfully managed without any complications during the perioperative period.

## Conclusions

In conclusion, we were able to avoid hemodynamic instability and to reduce opioid dosage in a centenarian who underwent emergency LC under general anesthesia with subcostal TAPB.

## Abbreviations

LC: Laparoscopic cholecystectomy
TAPB: Transversus abdominis plane block
